# Relationship Between Emotional Intelligence, Self-Acceptance, and Positive Coping Styles Among Chinese Psychiatric Nurses in Shandong

**DOI:** 10.3389/fpsyg.2022.837917

**Published:** 2022-03-18

**Authors:** Qinghua Lu, Bin Wang, Rui Zhang, Juan Wang, Feifei Sun, Guiyuan Zou

**Affiliations:** ^1^Department of Nursing, Shandong Mental Health Center, Jinan, China; ^2^Department of Psychology, Shandong Provincial Hospital, Jinan, China; ^3^School of Nursing, Shandong First Medical University and Shandong Academy of Medical Sciences, Jinan, China; ^4^School of Public Health, Weifang Medical University, Weifang, China; ^5^Department of Psychology, Shandong Mental Health Center, Jinan, China; ^6^Shandong Mental Health Center, Jinan, China

**Keywords:** emotional intelligence, self-acceptance, positive coping styles, psychiatric nurses, China

## Abstract

**Background:**

Nurses are facing increasing pressure due to the progressing of society, broadening of nursing service connotation, and increasing of the masses’ demand for medical treatment. Psychiatric nurses face suicides, violence, and lost along with other accidents involving patients with mental disorders under higher psychological pressure. A coping style, which is affected by individual emotions and cognition, is an essential psychological resource that allows individuals to regulate stress. The purpose of this study was to investigate the correlation between self-acceptance and the positive coping style of psychiatric nurses, and investigate the mediating role of emotional intelligence.

**Methods:**

A total of 813 psychiatric nurses from six natural regions in Shandong Province were investigated using the Self-Acceptance Questionnaire (SAQ), Emotional Intelligence Scale (EIS), Simplified Coping Style Questionnaire (SCSQ), and self-compiled general information questionnaire.

**Results:**

The total EIS score of psychiatric nurses was 3.848 ± 0.459. The highest score was for others’ emotional management (4.071 ± 0.548) and the lowest was for emotion perception (3.684 ± 0.483). EIS and positive coping style were statistically significant based on age, work experience, professional title, education level, and gender (*p* < 0.05, *p* < 0.01). Self-acceptance was statistically significant only for professional titles (*F* = 3.258, *p* = 0.021). Self-acceptance and emotional intelligence were positively correlated with positive coping style (*r* = 0.361, *p* < 0.01; *r* = 0.492, *p* < 0.01, respectively). The factors were also positively correlated with each other (*r* = 0.316, *p* < 0.01). Self-emotion management, others’ emotional management, emotion perception, self-acceptance, and education level jointly predicted positive coping styles (*R^2^* = 0.305, *F* = 60.476, *p* = 0.000). Emotional intelligence partially mediated the relationship between self-acceptance and positive coping styles, with a mediating effect of 16.3%.

**Conclusion:**

Emotional intelligence and self-acceptance can promote positive coping styles and improve psychiatric nurses’ mental health.

## Introduction

A coping style is a habitual tendency to solve problems or a common strategy to deal with stressors. It involves cognitive and behavioral strategies that individuals used to manage the internal and external demands of stressful events (Wang et al., 2007). Today, China is in a period of rapid development, and the pace of life is constantly accelerating. People face various unavoidable stressful situations, which influence their psychology. Nurses face increasing medical treatment demands that affect all areas of their personal and professional lives, and increase their risk of chronic stress and unhealthy behaviors (Tsaras et al., 2018). Psychiatric nurses face more suicide, violence, lost along with other accidental risks of patients with mental disorders while bearing high mental pressure. However, the same stress level affects individuals differently, mainly depending on their coping mechanism. Coping is a complex and multidimensional attitude and behavior process involving multiple strategies. The attitude and specific behavior (used to solve the problem actively or avoid it) affect the consequences of stressful situations. These differences in cognition, attitude, and behavior constitute diverse coping styles in stressful situations. Previous studies have demonstrated that the regular use of positive coping styles can change cognition, produce positive emotions and behaviors, improve job satisfaction, and relieve mental fatigue (Peng et al., 2013). They can also help individuals gain more support, reduce negative emotions, increase self-confidence, and improve self-efficacy and work efficiency (Zhang et al., 2016). Conversely, people who use more passive coping styles tend to doubt their abilities and have poorer mental health, reducing their quality of life (Li et al., 2021a). Therefore, psychiatric nursing staff should be encouraged to adopt a positive coping style, and influencing factors should be further investigated.

Studies have identified several factors associated with positive coping styles, including psychological resilience (Wu et al., 2020), self-esteem (Li et al., 2020), emotional intelligence (Fteiha and Awwad, 2020), and self-acceptance (Zeng, 2005). Self-acceptance is defined as respect or a positive attitude toward oneself as a whole, including one’s past experiences. Recognition of self-acceptance does not depend on the achievements of other individuals (Ellis, 2005). Self-acceptance is an important sign of mental health and a prerequisite for getting along well (Carson and Langer, 2006). According to Folkman et al. (1986), stress-and-coping mode occurs when individuals perceive a negative event (e.g., discrimination) as stressful and a threat to their self-image. This threat may significantly predict an individual’s self-acceptance, which may be directly related to their mental health. It has been proposed that self-acceptance is self-evaluation and the resulting self-experience and attitudes (Li et al., 2021b). Individuals with good self-acceptance can present themselves more truthfully in social situations, are conducive to more effective interpersonal interactions, have higher self-worth, and experience less loneliness. In addition, Han et al. (2016) found that individuals with high self-acceptance tend to use positive coping styles. Cao et al. (2014) found that self-acceptance was closely related to mental health and had a certain predictive effect on coping styles. The higher the degree of self-acceptance, the more inclined individuals are to adopt positive coping styles. The self-acceptance attitude encourages nurses to be more active and confident in their communication with doctors and patients so that they are more easily accepted and can establish a good interpersonal relationship (Wu et al., 2011). In psychiatry, although reducing the stigma of mental disorders has been of great importance in recent years, the public has generally continued to believe that patients with mental disorders are dangerous, unpredictable, and violent, thereby adopting a discriminatory attitude toward them (Zhang et al., 2009). Psychiatric nurses, as caregivers of patients with mental disorders during hospitalization, may overprepare themselves due to a feeling that they “have to understand the feelings of patients” or “should feel empathy with patients”; consequently, they may abandon or try to ignore their own feelings, leading to psychological distress and emotional reactions (Cao et al., 2015). The acceptance of uncontrollable negative events and the emotions that they elicit has been found to be protective at both psychological (decreasing negative emotions) and physical (providing immunity and decreasing pain) levels (e.g., Burns et al., 2002; McCracken and Eccleston, 2003). Therefore, the status quo of self-acceptance and its relationship with the coping style of psychiatric nurses are worth studying.

Emotional intelligence is the ability to monitor moods and emotions and use that information to guide thoughts and behaviors. It comprises perceiving, understanding, managing, and using emotions. Perceiving emotions is described as the ability to recognize one’s emotions when they occur. Understanding emotions is the core of emotional intelligence. It is the ability to understand and experience the emotions of others. In addition, it is the source of self-awareness and altruism, and the basis of moral judgment and action. Managing emotions is the ability to control emotions for oneself restoring in psychological frustration, restoring time from joy or anger to a normal state, and controlling one’s emotions without getting excited. Using emotions is the ability to utilize emotions to improve performance and control emotions positively and productively (Schutte et al., 1998). Emotional intelligence has been recognized as the most important social psychological concept. It is regarded as one of the crucial elements of a successful life and psychological well-being (Bar-On, 2010). It was primarily explained by Salovey et al. (1990) as the competency of possessing emotional knowledge, perceiving and controlling emotions well, and stimulating intellectual and emotional growth. Afterward, emotional intelligence was described as the capability to observe feelings, coordinate feelings, understand and control feelings, and stimulate self-improvement (Mayer and Salovey, 1997). Goleman (1995) characterizes emotional intelligence as skills or capabilities, including continuation despite hindrances, dealing with impulse and dissatisfaction, managing one’s mindsets, and keeping sufferings from influencing the capability to think, sympathize, and be hopeful. His emotional intelligence framework encompasses five key areas: self-awareness, self-regulation, social skills, motivation, and empathy. Bradberry and Greaves (2009) expressed that emotional intelligence includes interpersonal and intrapersonal intelligence. Interpersonal intelligence is the external intelligence an individual utilizes to understand and maintain relations with other individuals. It is imperative for promoting characteristics like sympathy and empathy, and for strengthening powerful relationships. Intrapersonal intelligence is the internal intelligence used by an individual to understand the self, which is necessary for self-awareness, self-inspiration, and self-regulation. Individuals with high emotional intelligence are expected to regulate, understand, and control their emotions and those of other individuals. People with high emotional intelligence tend to be sociable and well-perceived, and those with high emotional control tend to enjoy interacting with others. Individuals with high emotional intelligence are better equipped to select effective emotional regulation strategies than those with low emotional intelligence (Fletcher et al., 2012). Studies have indicated that emotional intelligence and stress-coping strategies significantly affect self-efficacy (Morales-Rodríguez and Pérez-Mármol, 2019). According to Epstein (1998), emotionally intelligent children are healthier, happier, and more adaptable, and these traits lead to desired academic achievements. Several studies have shown a significant correlation between emotional intelligence and coping style. Albesher and Alsaeed (2015) suggested that an increase in emotional intelligence increases the use of positive coping methods. A lower emotional intelligence level is related to suicidal and criminal behavior, not conducive to individual physical and mental health (Sharma et al., 2015; Domínguez-García and Fernández-Berrocal, 2018; Obeid et al., 2021). Studies have found that teens with high emotional intelligence maintain better mental health by adopting effective positive coping strategies (Davis and Humphrey, 2012). The ability to self-manage emotions is a critical factor in stressful situations and overall emotional management (Stelnicki et al., 2015; Fitzpatrick, 2016). Other studies have shown that high emotional intelligence is considered a prerequisite for building good social relationships because it can enable appropriate identification of feelings in oneself and others, demonstration of empathy, and effective regulation of emotions (Frederickson et al., 2012).

Emotional intelligence, as a kind of emotional operation ability, not only promotes individuals’ self-recognition and self-evaluation but also improves their self-acceptance and job satisfaction (Suleman et al., 2020). Studies have indicated that emotional intelligence and stress-coping strategies significantly affect self-efficacy (Morales-Rodríguez and Pérez-Mármol, 2019). A multinational study confirmed that high emotional intelligence was positively associated with high self-esteem, self-efficacy, and self-acceptance (Bhullar et al., 2013). Emotional intelligence has also been shown to be related to psychological health across different samples. Individuals with high emotional intelligence can better identify and regulate their own emotions, easily recover after being disturbed by stimuli, use psychological defense mechanisms effectively, change cognition, have more self-acceptance, and influence individual behavior patterns. As emotional intelligence is known to be improved by learning, experience, and training (Gardner, 1995), finding ways to enhance employees’ ability to control their emotions and empathize will help them improve their emotional intelligence. The aspects of emotional intelligence that play a greater role in the relationship between self-acceptance and coping styles are unclear. Therefore, it is necessary to explore the mediating mechanism of emotional intelligence (Cheng et al., 2020).

This study aimed to examine the correlation between self-acceptance, positive coping styles, and emotional intelligence among psychiatric nurses. Furthermore, it attempted to verify the mediating role of emotional intelligence between self-acceptance and positive coping style to provide a theoretical basis for nursing management, improve the emotional management ability of psychiatric nurses, and optimize coping styles.

## Materials and Methods

### Participants

This study was cross-sectional and used stratified sampling. The researchers divided Shandong province in China into six regions and randomly selected a tertiary psychiatric hospital from each region. The cluster sampling method was adopted to select psychiatric nurses from six psychiatric hospitals in the study. Participants who were included had: (1) obtained the National Qualified Certificate of Practice Nursing; (2) at least 1 year of clinical nursing work in a psychiatric department; and (3) volunteered to participate in this survey. Exclusion criteria included the following: (1) nursing interns; (2) nurses who had been out of clinical work for more than 3 months; and (3) refusal to participate in this survey. A total of 930 psychiatric nurses were included in the study.

### Questionnaire Collection Procedure

The researchers selected full-time investigators after communicating with the director of the nursing department of the selected hospitals. Full-time investigators issued paper questionnaires and explained the filling method and key points to psychiatric nurses who participated in the survey. Before the questionnaire survey, the informed consent of the subjects was obtained. The researcher informed the subjects in detail that the data from this study would be used for research and followed the principle of confidentiality. The subjects were also informed that there was no need to provide personal identity information, such as their name or work number; they only needed to answer truthfully according to their situation; and the results would be returned within 1 week. A total of 894 questionnaires were collected, of which 813 were valid. The effective response rate was 90.9%.

### Ethical Considerations

The study was approved by the Ethics Committee of the Shandong Mental Health Center. This survey strictly followed the informed consent principle and strictly protected participants’ right to anonymity and privacy. The participants provided written informed consent when completing the questionnaires.

### Questionnaire Design and Reliability and Validity Tests

Based on the literature reading, a structured questionnaire design was proposed. Fifty psychiatric nurses were randomly selected from a provincial mental health center for the questionnaire survey to ensure the reliability of the questionnaire. Two weeks later, these nurses were asked to retake the questionnaire, and comparative analysis showed that the retest coefficient was 0.82, indicating that the questionnaire had high reliability. The questionnaire comprised the following four parts.

#### Measurement of General Information

The general information questionnaire included age, gender, length of service, education background, marital status, and professional title.

#### Measurement of the Self-Acceptance Questionnaire

The Self-Acceptance Questionnaire (SAQ) includes 16 items introduced by Chinese scholars (Cong and Gao, 1999). This questionnaire was used to assess self-acceptance characteristics. The items included two factors: self-acceptance (SA) and self-evaluation (SE). Each factor comprised eight items. SE was scored positively and rated on a 4-point Likert scale ranging from four (significantly similar) to one (significantly opposite). SA was scored in reverse and rated from one (significantly similar) to four (significantly opposite). The total score for each factor was calculated as the cumulative score of each item. The total SAQ score was the cumulative score of each factor. The higher the score, the higher the degree of self-acceptance. Cronbach’s α was 0.810 and 0.781 for the subscales.

#### Measurement of Emotional Intelligence Scale

The Emotional Intelligence Scale (EIS) was compiled by American psychologists Schutte et al. in 1998, according to the emotional intelligence theory of Mayer and Salovey (1997); the scale included four dimensions and 33 items (Schutte et al., 1998). The Chinese version was translated by Wang, who also demonstrated that EIS has good reliability and validity (Wang, 2002). EIS involved emotion perception (12 items), self-emotion management (nine items), others’ emotional management (six items), and emotion application (five items). Each item was rated on a 5-point Likert scale ranging from one (significantly inconsistent) to five (significantly consistent). Items 5, 28, and 33 were scored in the reverse. The Cronbach’s α of the four dimensions in this study were 0.851, 0.725, 0.710, and 0.568.

#### Measurement of Simplified Coping Style Questionnaire

The Simplified Coping Style Questionnaire (SCSQ) was compiled by Xie (1998); it includes two dimensions and 20 items. Its reliability and internal validity were verified. The positive coping style subscale comprised 12 items (1–12), measuring the characteristics of positive coping. Example statements for this factor include “try to realize the bright side” and “find different ways to solve the problem.” The negative coping style subscale consisted of eight items (13–20) that measured the characteristics of negative coping. Example statements for this factor include “relieve troubles by smoking and drinking” and “fantasize that the situation would change miraculously.” Each item was rated on a 4-point Likert scale ranging from 0 (never use) to three (often use). Cronbach’s *α* was 0.842 and 0.754 for the subscales.

### Statistical Analysis

Data input and statistical analysis were conducted using SPSS 22.0 and AMOS 22.0, and statistical significance was set at *p* < 0.05. Continuous variables were expressed as mean and standard deviation, categorical variables were expressed as frequency and percentage, and univariate analysis was performed by t-test or single-factor chi-square test to assess the sample characteristics and relationships among variables. Pearson correlation, multiple-step regression, structural equation modeling, R 4.1.2, and nonparametric percentile bootstrap test were used to analyze the relationships among self-acceptance, emotional intelligence, and coping style.

## Results

### General Information for Psychiatric Nurses

The 813 psychiatric nurses were between 19 and 56 years old, with an average age of 30.84 ± 7.94. Their experience in psychiatric nursing ranged between 1 and 40 years. The median work experience was 5 years (*p*25 = 2 years, *p*75 = 15 years). Overall, 187 respondents were male (23.0%), and 626 were female (77.0%). Regarding educational background, 447 (55.0%) had a college degree or below, and 366 (45.0%) had a bachelor’s degree or above. Moreover, 516 were married (63.5%), and 397 were not (36.5%). Regarding professional titles, 366 were nurses (45.0%), 245 were junior nurses (30.1%), 166 were senior nurses (20.42%), and 36 were associate superintendent nurses (4.23%).

### Status of Emotional Intelligence, Self-Acceptance, and Coping Styles Among Psychiatric Nurses

The total EIS score of psychiatric nurses was 3.848 ± 0.459, and the scores of each dimension ranging from high to low were 4.071 ± 0.548, 3.956 ± 0.537, 3.812 ± 0.546, and 3.684 ± 0.483 for others’ emotional management, self-emotion management, emotion application, and emotion perception, respectively. The total SAQ score was 42.28 ± 4.91, the SA score was 21.76 ± 3.60, and the SE score was 20.53 ± 3.36. Compared with the domestic norm (Cong and Gao, 1999), there was no statistically significant difference in SAQ (*t* = 0.61, *p* > 0.05), SA (*t* = 0; *p* > 0.05), and SE (*t* = 0.97; *p* > 0.05). The score of positive coping style was 2.09 ± 0.47; there was a statistically significant difference compared with the domestic norm of 1.78 ± 0.52 (*t* = 12.76, *p* = 0.000; Xie, 1998).

### Characteristics of the Participants and the Comparisons of the Scores on Emotional Intelligence, Self-Acceptance, and Positive Coping Styles

Regarding positive coping styles, there was a significant difference among nurses, junior nurses, and associate superintendent nurses (*F* = 3.101, *p* = 0.026). Psychiatric nurses with a bachelor’s degree or above scored higher than those with a college degree or below (*F* = −3.842, *p* = 0.000). Females scored higher than males (*F* = −2.003, *p* = 0.046).

Regarding scores on others’ emotional management (*F* = 4.564, *p* = 0.011), psychiatric nurses working for 10 years or more scored higher than those less experienced (*p* < 0.05), and nurses over 35 years old scored higher than those under 35 years of age (*p* < 0.01). Regarding scores on self-emotion management, psychiatric nurses over 35 years old scored higher than those under 35 years old (*F* = 4.662, *p* = 0.010), associate superintendent nurses scored higher than other nurses (*F* = 4.223, *p* = 0.006), and females scored higher than males (*t* = −1.987, *p* = 0.048). Regarding scores on emotion perception, psychiatric nurses with a bachelor’s degree or above scored higher than those with a college degree or below (*t* = −2.967, *p* = 0.003), and associate superintendent nurses scored higher than other nurses (*F* = 4.269, *p* = 0.005). Regarding scores on the SAQ, associate superintendent nurses scored higher than nurses, junior nurses, and senior nurses (*F* = 3.258, *p* = 0.021; Table 1).

**Table 1:** Characteristics of the participants and the comparisons of the scores on emotional intelligence, self-acceptance, and positive coping styles (*n* = 813).

	Variables	*n*	Positive coping styles	Emotion perception	Self-emotion management	Emotional management of others	Emotion application	SAQ
Length of service	<5 years	409	24.91 ± 5.60	3.66 ± 0.49	3.95 ± 0.54	4.03 ± 0.56^**^	3.81 ± 0.56	42.37 ± 4.86
	5–10 years	156	24.45 ± 5.67	3.70 ± 0.45	3.90 ± 0.53^*^	4.04 ± 0.54^*^	3.79 ± 0.52	42.10 ± 4.49
	>10 years	248	25.60 ± 5.81	3.71 ± 0.50	4.00 ± 0.53	4.16 ± 0.52	3.82 ± 0.55	42.26 ± 5.27
	*F*		2.154	1.002	1.904	4.564	0.216	0.174
	*p*		0.117	0.368	0.150	0.011	0.806	0.840
Age	≤25	257	24.83 ± 5.40	3.67 ± 0.49	3.97 ± 0.55	4.03 ± 0.55^**^	3.82 ± 0.54	42.12 ± 5.09
	26–35	351	24.78 ± 5.63	3.66 ± 0.47^*^	3.90 ± 0.53^**^	4.03 ± 0.55^**^	3.78 ± 0.55	42.31 ± 4.50
	>35	205	25.72 ± 6.10	3.74 ± 0.50	4.04 ± 0.52	4.18 ± 0.53	3.85 ± 0.54	42.44 ± 5.37
	*F*		2.013	2.166	4.662	5.826	1.062	0.776
	*p*		0.134	0.115	0.010	0.003	0.346	0.254
Professional title	Nurse	366	25.00 ± 5.74^*^	3.67 ± 0.48^**^	3.98 ± 0.56^*^	4.06 ± 0.57	3.83 ± 0.57	42.54 ± 4.99^*^
	Junior nurse	245	24.39 ± 5.49^**^	3.64 ± 0.48^**^	3.88 ± 0.49^**^	4.02 ± 0.50^*^	3.76 ± 0.50	41.80 ± 4.35^**^
	Senior nurse	166	25.64 ± 5.77	3.71 ± 0.48^**^	3.98 ± 0.56^*^	4.15 ± 0.56	3.81 ± 0.57	42.00 ± 4.97^*^
	Associate superintendent nurse	36	26.97 ± 5.61	3.94 ± 0.48	4.19 ± 0.48	4.24 ± 0.55	3.94 ± 0.54	44.25 ± 6.69
	*F*		3.101	4.269	4.223	2.924	1.526	3.258
	*p*		0.026	0.005	0.006	0.033	0.206	0.021
Education background	College degree or below	447	24.35 ± 5.84	3.64 ± 0.48	3.94 ± 0.56	4.04 ± 0.56	3.81 ± 0.55	42.36 ± 4.98
	Bachelor’s degree or above	366	25.87 ± 5.38	3.74 ± 0.48	3.98 ± 0.50	4.11 ± 0.53	3.81 ± 0.54	42.18 ± 4.85
	*t*		-3.842	−2.967	−1.005	−1.843	0.004	0.524
	*p*		0.000	0.003	0.315	0.066	0.997	0.600
Gender	Male	187	24.30 ± 5.98	3.64 ± 0.51	3.89 ± 0.56	4.00 ± 0.58	3.80 ± 0.55	42.34 ± 4.47
	Female	626	25.25 ± 5.58	3.70 ± 0.47	3.98 ± 0.53	4.09 ± 0.54	3.82 ± 0.55	42.27 ± 5.04
	*t*		−2.003	−1.276	−1.987	−1.960	−0.485	0.171
	*p*		0.046	0.203	0.048	0.051	0.628	0.864
Marital status	Single	297	25.07 ± 5.45	3.68 ± 0.47	3.98 ± 0.53	4.03 ± 0.56	3.84 ± 0.56	42.05 ± 4.89
	Married	516	25.01 ± 5.82	3.69 ± 0.49	3.94 ± 0.54	4.09 ± 0.54	3.80 ± 0.54	42.42 ± 4.93
	*t*		0.151	−0.090	1.124	−1.539	1.152	−1.023
	*p*		0.880	0.928	0.261	0.124	0.250	0.307

Work experience: compared with “>10 years,” ^*^p < 0.05; ^**^p < 0.01; Age: compared with “>35 years,” ^*^p < 0.05; ^**-^*p* < 0.01; Professional title: compared with “Associate superintendent nurse,” ^*^p < 0.05, ^**^p < 0.01.

### Relationship Between EIS, SAQ, and SCSQ Among Psychiatric Nurses

**Figure 1:**
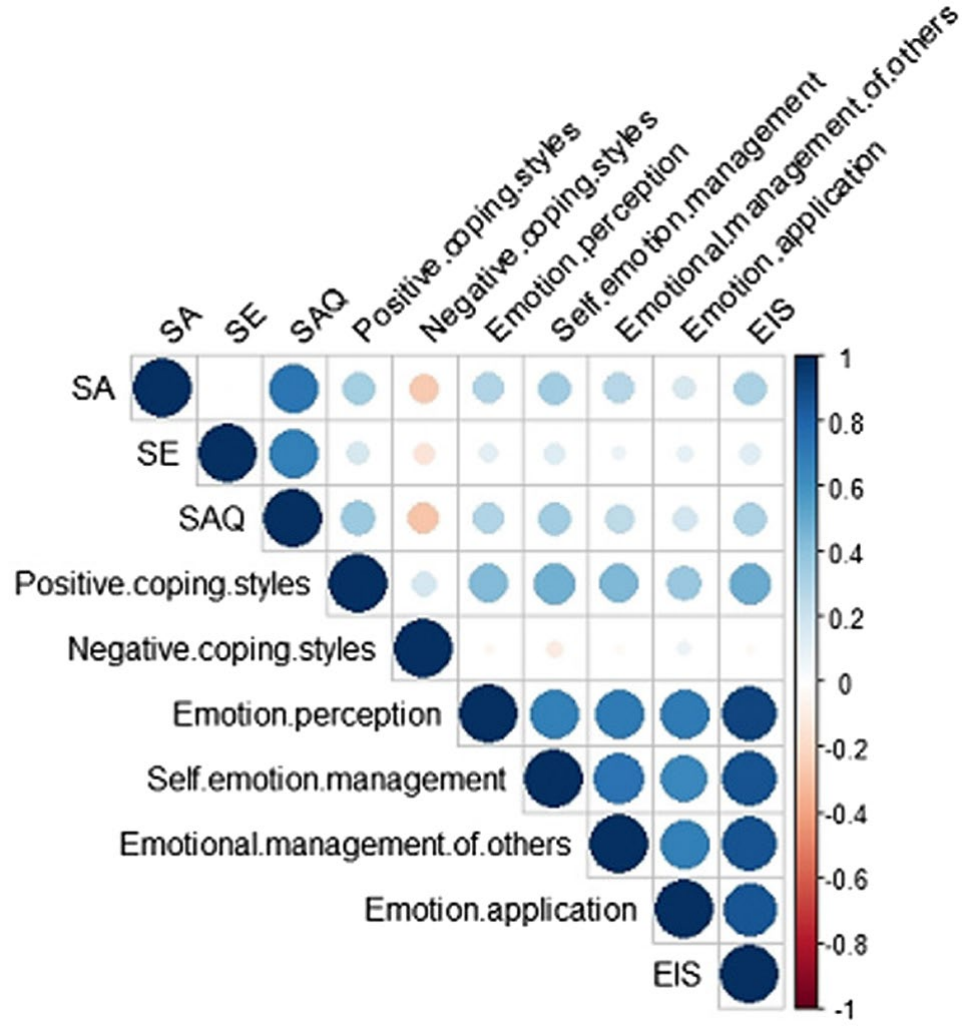
Heat map of the relationship between Emotional Intelligence Scale (EIS), Self-Acceptance Questionnaire (SAQ), and Simplified Coping Style Questionnaire (SCSQ) among psychiatric nurses. SA, self-acceptance and SE, self-evaluation.

The SAQ was positively correlated with positive coping styles (*r* = 0.361, *p* < 0.01) and negatively with negative coping styles (*r* = −0.279, *p* < 0.01). EIS was positively correlated with SAQ (*r* = 0.316, *p* < 0.01) and positive coping styles (*r* = 0.492, *p* < 0.01). Self-emotion management (*r* = −0.096, *p* < 0.01) was negatively correlated with negative coping styles. (Table 2). In order to express the correlation of various indicators more clearly, R 4.1.2 was used to draw the correlation heat map, as shown in Figure 1.

**Table 2:** Relationship between EIS, SAQ, and SCSQ among psychiatric nurses.

Variables	1	2	3	4	5	6	7	8	9	10
1. SA	1									
2. SE	−0.003	1								
3. SAQ	0.730^**^	0.681^**^	1							
4. Positive coping styles	0.340^**^	0.164^**^	0.361^**^	1						
5. Negative coping styles	−0.257^**^	−0.134^**^	−0.279^**^	0.178^**^	1					
6. Emotion perception	0.298^**^	0.119^**^	0.299^**^	0.433^**^	−0.034	1				
7. Self-emotion management	0.342^**^	0.138^**^	0.344^**^	0.477^**^	−0.096^**^	0.689^**^	1			
8. Emotional management of others	0.276^**^	0.079^*^	0.256^**^	0.445^**^	−0.028	0.701^**^	0.736^**^	1		
9. Emotion application	0.167^**^	0.097^**^	0.189^**^	0.374^**^	0.079^*^	0.709^**^	0.641^**^	0.687^**^	1	
10. EIS	0.313^**^	0.126^**^	0.316^**^	0.492^**^	−0.026	0.910^**^	0.870^**^	0.868^**^	0.855^**^	1
Cronbach’s α coefficient	0.810	0.781	0.738	0.842	0.754	0.851	0.725	0.710	0.568	0.908

** *p* < 0.01;

* *p* < 0.05.

### Analysis of Factors Associated With Positive Coping Styles of Psychiatric Nurses

A multiple stepwise regression analysis was used to analyze the associated factors of positive coping styles on psychiatric nurses (Table 3). Positive coping style was used as the dependent variable, and general demographic data of psychiatric nurses, self-acceptance, self-evaluation, emotional perception, self-emotional management, others’ emotional management, and emotional utilization were used as independent variables. Self-emotion management (*β* = 0.213, *p* = 0.000), self-acceptance (*β* = 0.199, *p* = 0.000), others’ emotional management (*β* = 0.151, *p* = 0.001), self-evaluation (*β* = 0.118, *p* = 0.000), education background (*β* = 0.109, *p* = 0.000), and emotion perception (*β* = 0.097, *p* = 0.000) were the associated factors of positive coping style (*R^2^* = 0.305, *F* = 60.476, *p* = 0.000).

**Table 3:** Multiple stepwise regression analysis of factors influencing positive coping styles in psychiatric nurses.

Variables	*B*	*SE*	*β*	*t*	*p*	Tolerance	VIF
(Constant)	−7.682	1.854	−	−4.144	0.000	−	−
Self-emotion management	0.281	0.063	0.213	4.500	0.000	0.383	2.613
Self-acceptance	0.314	0.050	0.199	6.339	0.000	0.871	1.147
Other’s emotion management	0.261	0.081	0.151	3.209	0.001	0.386	2.593
Self-evaluation	0.200	0.050	0.118	3.973	0.000	0.970	1.031
Educational background	0.972	0.262	0.109	3.708	0.000	0.981	1.019
Emotion perception	0.095	0.044	0.097	2.181	0.029	0.433	2.308

*R*^2^ = 0.310, Adjusted *R*^2^ = 0.305, *F* = 60.476, *p* = 0.000.

In order to further verify the effect of gender interaction on psychological scores according to gender stratification, we conducted a regression analysis to analyze the associated factors on 187 male and 626 female psychiatric nurses. Male group results showed that others’ emotional management (*β* = 0.445, *p* = 0.000), self-acceptance (*β* = 0.290, *p* = 0.000), and educational background (*β* = 0.129, *p* = 0.027) were the associated factors of positive coping style (*R^2^* = 0.399, *F* = 40.468, *p* = 0.000; Table 4).

**Table 4:** Multiple stepwise regression analysis of factors influencing positive coping styles in male psychiatric nurses.

Variables	*B*	*SE*	*β*	*t*	*p*	Tolerance	VIF
(Constant)	−6.281	2.948	−	−2.131	0.034	−	−
Other’s emotion management	4.627	0.670	0.445	6.909	0.000	0.791	1.265
Self-acceptance	0.439	0.097	0.290	4.510	0.000	0.796	1.257
Educational background	1.239	0.555	0.129	2.233	0.027	0.989	1.011

*R^2^ = 0.399, Adjusted R^2^ = 0.389, F = 40.468, P = 0.000.*

Female group results showed that self-emotion management (*β* = 0.205, *p* = 0.000), self-evaluation (*β* = 0.166, *p* = 0.000), self-acceptance (*β* = 0.161, *p* = 0.000), emotion perception (*β* = 0.118, *p* = 0.019), education background (*β* = 0.124, *p* = 0.001), others’ emotional management (*β* = 0.122, *p* = 0.020), and marital status (*β* = −0.081, *p* = 0.026) were the associated factors of positive coping style (*R^2^* = 0.293, *F* = 36.548, *p* = 0.000; Table 5).

**Table 5:** Multiple stepwise regression analysis of factors influencing positive coping styles in female psychiatric nurses.

Variable	*B*	*SE*	*β*	*t*	*p*	Tolerance	VIF
(Constant)	−5.936	2.133	−	−2.783	0.006	−	−
Self-emotion management	2.159	0.559	0.205	3.861	0.000	0.407	2.456
Self-evaluation	0.277	0.058	0.166	4.801	0.000	0.961	1.040
Self-acceptance	0.259	0.057	0.161	4.512	0.000	0.893	1.119
Emotion perception	1.382	0.589	0.118	2.345	0.019	0.456	2.194
Educational background	1.089	0.320	0.124	3.403	0.001	0.868	1.153
Other’s emotion management	1.264	0.541	0.122	2.335	0.020	0.420	2.383
Marital status	−0.967	0.434	−0.081	−2.228	0.026	0.867	1.153

*R^2^ = 0.293, Adjusted R^2^ = 0.285, F = 36.548, P = 0.000.*

### The Mediating Role of Emotional Intelligence in Self-Acceptance and Positive Coping Styles

**Figure 2:**
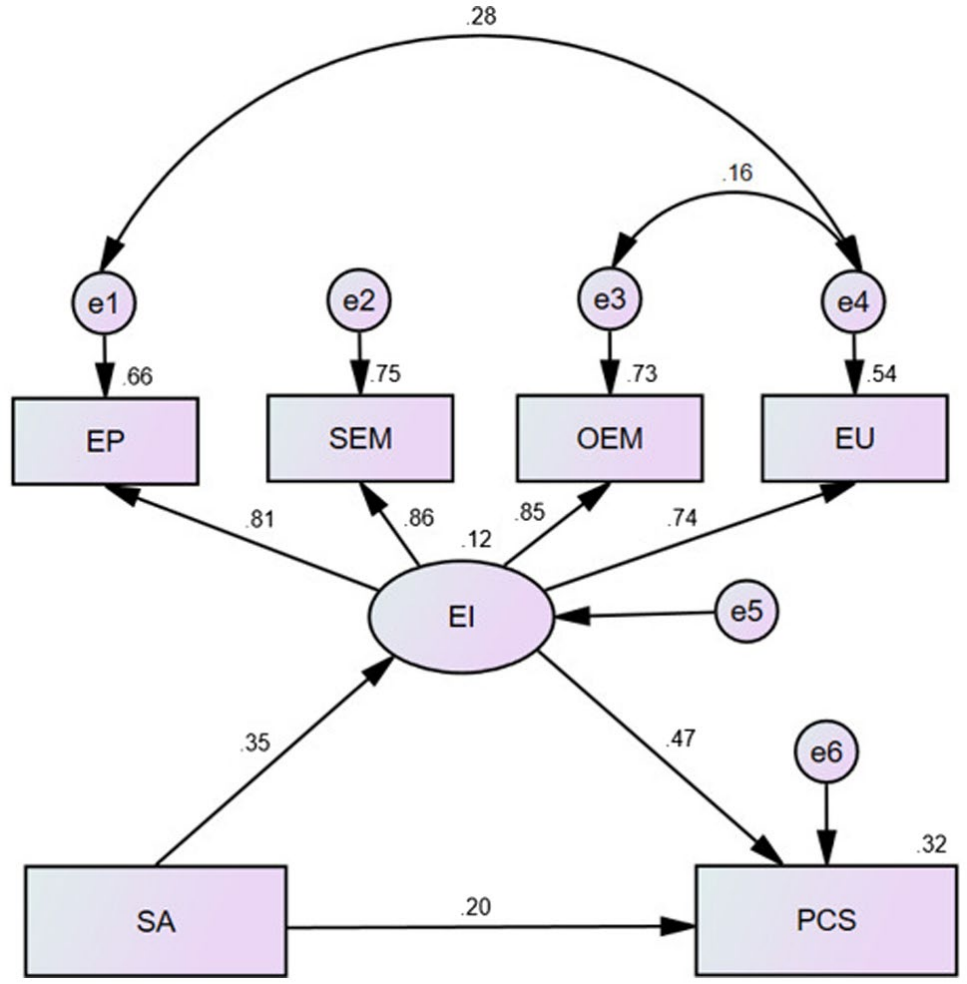
Standardized estimation of the relationships of emotional intelligence and self-acceptance with positive coping styles. SA, self-acceptance; EI, emotional intelligence; EP, emotional perception; SEM, self-emotion management; OEM, other’s emotion management; EU, emotion utilization; PCS, positive coping styles.

We constructed a structural equation model to further explore the relationships among emotional intelligence, self-acceptance, and positive coping styles (Figure 2; Table 6). Figure 2 shows the model of the relationship between emotional intelligence, self-acceptance, and positive coping styles. The original fitness of the model was indicated through the following parameters: χ^2^ = 62.952, RMSEA = 0.092, NFI = 0.974, CFI = 0.977, GFI = 0.975, AGFI = 0.934, and GMIN/DF = 7.869. These parameters indicated that the model was not suitable. After two adjustments, the model fit was good, as evidenced through the following parameters: χ^2^ = 27.006, RMSEA = 0.066, NFI = 0.989, CFI = 0.991, AGFI = 0.963, and GMIN/DF = 4.501; moreover, the difference in each path structure was statistically significant (*p* < 0.01). Table 6 shows the direct, indirect, and overall effects of emotional intelligence and self-acceptance on positive coping styles. The results showed that self-acceptance had a direct and indirect effect, whereas emotional intelligence had only a direct effect on positive coping styles. Among the four dimensions of emotional intelligence, the absolute value of self-emotion management was the highest (0.86), followed by others’ emotion management (0.85).

**Table 6:** The mediating role of emotional intelligence in self-acceptance and positive coping styles.

Variables	Total effect	Direct effect	Indirect effect
SA	0.361	0.198	0.163
EI	0.466	0.466	0.000

SA = self-acceptance and EI = emotional intelligence.

## Discussion

### Current Situation of Emotional Intelligence, Self-Acceptance, and Coping Styles Among Psychiatric Nurses

In this study, the average EIS score was 3.848 ± 0.459. The score was higher than that obtained in the Chinese survey of 204 nursing students, which was 3.63 ± 0.22 (*t* = 9.863, *p* = 0.000; Wei et al., 2012). Another survey of 108 psychiatric-mental health nurses (PMHNs) revealed significantly different scores than those obtained by the 5,000 respondents included in the normed sample. PMHNs in the study had a higher mean emotional intelligence compared with the 5,000 participants in the normed sample (Sims, 2017). The EIS scores of each dimension (ranging from high to low) were others’ emotional management, self-emotion management, emotion application, and emotion perception. This result is inconsistent with the internal structure of emotional intelligence in college students or nurses in general hospitals (Lawal and Idemudia, 2017; Wen et al., 2020). A study conducted among college students showed that the score of self-emotion management was the highest, and emotional perception was the lowest (Wen et al., 2020); additionally, the emotional intelligence scores of nurses in general hospitals were the highest for emotion application, followed by scores for emotion perception, self-emotion management, and others’ emotional management (Lawal and Idemudia, 2017). Psychiatric nurses scored the highest on others’ emotional management, perhaps due to their interactions with people with mental disorders and their families. Therefore, they may overprepare, as they must “understand others’ feelings” or “feel empathy for others.” In this process, they may abandon or try to ignore their feelings (Cunico et al., 2012). Psychiatric nurses scored the lowest on emotional perception. This low score could be because all the hospitals investigated in this study operate closed management wards in the psychiatric department. Therefore, patients cannot enter and exit the wards at will. The average length of stay in mental health hospitals is 50–60 days, which is usually higher than that in general hospitals (Han et al., 2021). The closed management mode of psychiatric hospitals leads to a lack of contact with the outside world, making the nursing staff prone to isolation and depression psychological defense mode. Moreover, most hospitals lack a work environment or emotional engagement team that can convey both positive and negative emotions, resulting in psychiatric nurses’ reduced ability to perceive emotions. As most patients with mental disorders have emotional, behavioral, and cognitive issues (along with other abnormal aspects), continuous engagement by the nurses to serve the patient’s psychiatric nurses may result in the nurses experiencing excessive emotions relating to the patient, consequently desensitizing them to the act of perceiving emotions. A Japanese study also confirmed that psychiatric nurses have significantly lower self-awareness than those in other fields (Ishii and Horikawa, 2019).

Self-acceptance is an important personality trait and is crucial for individual growth. This study showed that the SAQ score was not significantly different between psychiatric nurses and domestic norms. Hence, although psychiatric nurses are reluctant to work in psychiatric settings due to stressors associated with working in psychiatric settings, emotional burnout, and negative public attitudes toward psychiatric settings (Rahmani et al., 2021), studies have reported that most nurses choose to work in psychiatric hospitals only when there are no other employment opportunities (Atashzadeh-Shoorideh et al., 2018). For those engaged in psychiatric nursing, it does not affect the overall self-acceptance attitude.

The positive coping style score of psychiatric nurses was higher than the domestic norm, indicating that psychiatric nurses can realize the specificity of the work object and adopt a positive, enthusiastic, and positive attitude in communication with patients or their families.

### Association of Demographic Characteristics on Emotional Intelligence, Self-Acceptance, and Positive Coping Styles in Psychiatric Nurses

#### Age and Length of Service

This study showed statistical significance in the emotional intelligence scores of psychiatric nurses of different ages and years. Consistent with previous research (Alshammari et al., 2020), the older the nurse, the stronger their ability to perceive others’ emotions and manage their own. Additionally, nurses who have worked longer were also more skilled at understanding others’ emotions. As they have been working in psychiatric care for years, older psychiatric nurses may have gradually accumulated work experience and improved their emotional intelligence. This result also indicates that emotional intelligence is a skill that can be acquired through teaching and learning, and is related to practice and experience (Mayer et al., 2000). With the increase in age and work experience, psychiatric nurses could more accurately identify patients’ psychotic symptoms; perceive the patients’ mood changes; and use empathy, listening, affirmation, and other communication skills to deal with these bad moods. These experiences improved their emotional intelligence and communication skills and enabled them to face and deal with stress more positively (Molero Jurado et al., 2019).

#### Professional Titles

There were statistically significant differences in emotional intelligence, coping style, and self-acceptance scores among psychiatric nurses with different professional titles. Compared with primary and intermediate nurses, senior psychiatric nurses scored higher on emotional perception, self-emotional management, and others’ emotional management. Regarding the current standards for the promotion of nurses’ professional titles, there are many important conditions, such as the nurses’ educational background, work experience, workload, and scientific research level (Zhao, 2009). The professional title represents the ability and level of nursing staff and comprehensively reflects the nurses’ professional theory, technical level, and scientific research ability. In this study, those with senior professional titles generally worked for a relatively long time and engaged in nursing management in the ward. They needed to conduct more communication, coordination, and business guidance and solve various conflicts in their work, which further improved their abilities comprehensively (Happell et al., 2020).

#### Educational Background

Consistent with a previous study (Hu et al., 2010), nurses with bachelor’s degrees or above scored higher in emotional perception ability and positive coping style than other nurses. Most undergraduate nursing colleges and universities conducted “nursing psychology,” “nursing aesthetics,” “nursing interpersonal communication,” and other courses (Casey, 2009), which helped improve the nurses’ interpersonal communication skills and confidence. Meanwhile, those with higher education levels were more engaged in technical and managerial nursing work, which strengthened their holistic abilities and helped them to deal with emergencies more effectively.

#### Gender

Our study showed that female nurses scored higher than male nurses on positive coping style, self-emotional management, and understanding of others’ emotions, consistent with the results of the study by Schutte et al. (1998). However, Erol et al. (2013) found that different genders have differences in emotion perception and processing. Women’s perception of negative emotions is more sensitive and accurate, even in the womb. Moreover, the nature of psychiatric care may also be an associated factor. Psychiatric nurses have a high incidence of workplace violence (Konttila et al., 2020). A study of conflict management models among Chinese psychiatric nurses demonstrated that male nurses tended to adopt a competitive mode during conflicts, whereas female nurses tended to adopt an accommodating mode (Lu and Zhong, 2015). This study also indicated that the latter were better at recognizing patients’ mental symptoms and emotional changes, and timely adjusting their emotions to cope with work conflicts.

### Correlation Between Emotional Intelligence, Self-Acceptance, and Coping Styles of Psychiatric Nurses

This study shows that emotional intelligence and self-acceptance are positively correlated with positive coping styles. Coping style is an individual’s cognitive or behavioral effort to reduce stress in stressful situations. It is an important variable that affects stress and the quality of life through physical and mental states (Labrague et al., 2018). Emotional intelligence is the ability to curb negative emotions of anger, low self-esteem, and anxiety and replace them with positive emotions such as confidence, empathy, and friendship (Lee et al., 2019). It is closely related to individual mental health and adaptation levels, and determines people’s behavior when facing problems. Wang and Xie (2016) conducted a study with 537 nurses from two Chinese universities that showed a significant relationship between emotional intelligence and active methods of coping with stress.

Bar-On (2010) showed that emotional intelligence has a significant impact on an individual’s capacity for positive social interaction. Shah and Thingujam (2008) suggested that a positive coping style is the foundation for good physical and mental health. Successful coping strategies lead to a balanced emotional response in highly stressful situations. Positive coping strategies (e.g., meditation, exercise, good nutrition, relaxation, humor, and fun-filled activities) can reduce stress (Ugoji, 2012). This study shows that psychiatric nurses with high emotional intelligence tend to adopt positive coping styles. In long-term psychiatric nursing, nurses often face sudden accidents, such as violence and suicide, along with patient escapes during an emergency. Nurses with a strong emotion management ability and a more stable and positive mood tend to meet the challenge and actively cope with it.

Self-acceptance was positively correlated with a positive coping style. Psychiatric nurses with high self-acceptance can objectively recognize and evaluate problems, accept and face themselves, and accurately recognize and change during problems, to better manage their emotions (Lindsay and Creswell, 2019). They will make rational analyses during problems and challenges and actively seek solutions, which is a positive coping style. However, psychiatric nurses with low self-acceptance levels are reluctant to face their shortcomings and deficiencies. When facing problems and challenges, they often adopt negative escape methods, as is consistent with previous research results (Paloș and Vîșcu, 2014; Faustino et al., 2020).

This study also showed that self-emotion management, self-acceptance, other’s emotion management, self-evaluation, education background, and emotion perception associated with positive coping styles. In order to further verify the effect of gender interaction on psychological scores according to gender stratification, we conducted a regression analysis to analyze the associated factors on 187 male and 626 female psychiatric nurses. The results showed that the factors associated positive coping styles were different in male and female populations. Among male psychiatric nurses, others’ emotional management, self-acceptance and educational background were the associated factors of positive coping style, while among female psychiatric nurses, self-emotional management, self-evaluation, self-acceptance, emotional perception, others’ emotional management, educational background, and marriage were the associated factors. Our data indicated that others’ emotional management, self-acceptance, and educational background were factors associated positive coping styles of both male and female psychiatric nurses, and that others’ emotional management and self-acceptance had higher predictive values for positive coping styles of male psychiatric nurses than for positive coping styles of female psychiatric nurses. Female positive coping style had more associated factors, but the *β* value was small. Self-emotional management, self-evaluation, emotional perception, and marriage predicted positive coping styles only for female psychiatric nurses. The above results indicate that gender has significant interaction on psychological scores in psychiatric nurses. The association of various variables on positive coping style had synergistic effects.

### Emotional Intelligence as a Mediator Between Self-Acceptance and Positive Coping Styles Among Psychiatric Nurses

Emotional intelligence had a statistically significant mediating effect between self-acceptance and coping styles. The bootstrap mediation test showed that the mediating effect was statistically significant, suggesting that self-acceptance directly affects positive coping styles and indirectly affects them through emotional intelligence. Self-acceptance is the inner self’s positive psychological performance toward a positive evaluation of self-acceptance, combining the internal and external aspects of the self. It is the starting point of mental health. It is theorized as acceptance of oneself despite weaknesses (Mearns, 1989), which is routinely shown to be related to positive outcomes (Chamberlain and Haaga, 2001). However, the inability to unconditionally accept oneself can lead to various emotional difficulties (e.g., uncontrolled anger and depression; Carson and Langer, 2006). Self-acceptance may suppress the effects of negative emotional experiences, thereby improving daily life experiences that lead to psychological distress. Psychiatric nurses improve their emotional management ability by changing their dissatisfaction and criticism, being too harsh on themselves, and timely self-counseling (Wu et al., 2018). They make rational analyses during problems and challenges, and actively seek solutions, which is a positive coping style (Xuan et al., 2017). Psychiatric nurses with high emotional intelligence employ it to work actively during pressure and show more positive emotional experiences to solve various problems, which enhances their self-acceptance and self-improvement and thereby enhances their ability to respond (Moshki et al., 2018). Psychiatric nurses with low emotional intelligence tend to adopt negative coping styles when they encounter conflicts and difficulties in their work, such as increased frustration and lack of self-confidence. Therefore, emotional intelligence is the mediating factor of self-acceptance, influencing a positive coping style.

### Limitations

This study had some limitations. First, this study was only conducted in six psychiatric hospitals in Shandong Province, China, without involving other provinces and cities; thus, the representativeness may be limited. Second, this was a cross-sectional study; therefore, no causal link could be established between the variables; The majority of participants in this study were female psychiatric nurses, and few male participants were included. In addition, this study used self-rating scales, and the results were self-reported by participants, due to which bias may have occurred. A national longitudinal study of self-acceptance, emotional intelligence, and coping styles among psychiatric nurses could be considered.

### Implications for the Theory and Practice

This study investigated the relationship between emotional intelligence and self-acceptance and positive coping style among psychiatric nurses in six tertiary psychiatric hospitals in Shandong Province, China. In order to guide the clinical nursing practice of psychiatry better, we should further expand the research field and carry out relevant intervention research.

1. Emotional intelligence develops gradually in the process of socialization. Nursing managers should be encouraged to continue education courses related to nursing aesthetics, interpersonal communication skills, negative emotion management, and conflict coping skills, and participate in group training projects. These will enhance psychiatric nurses’ self-emotion management ability, improve the nurses’ ability to adjust their negative emotions, and promote a harmonious relationship between nurses and patients, thereby improving psychiatric nurses’ mental health.
2. Self-acceptance, as an important part of personality and the social need for individual survival, has a certain correlation and predictive function with coping styles. It is suggested that group counseling courses such as acceptance and commitment therapy should be set up in the continuing education and training programs for psychiatric nurses to improve their self-acceptance level and help psychiatric nurses adopt positive coping styles to cope with pressure and frustration.
3. In the future, we should further explore the relationship between emotional intelligence, emotional regulation, psychological distress, and other psychological variables of psychiatric nurses, analyze their mutual influence and regulation mechanism, and carry out interpersonal relationship therapy, mindfulness-based stress reduction therapy, and emotional management ability intervention projects to improve the mental health level of psychiatric nurses.

## Conclusion

1. The emotional intelligence of Chinese psychiatric nurses is above the medium level, and there are differences based on age, work experience, professional title, educational background, and gender. There is no difference between the self-acceptance level of psychiatric nurses and domestic norms. Psychiatric nurses are more likely to adopt a positive coping style.
2. The emotional intelligence of psychiatric nurses was positively correlated with self-acceptance and positive coping styles. Emotional intelligence, self-acceptance, and educational background predicted positive coping styles.
3. Emotional intelligence partially mediated the relationship between self-acceptance and positive coping styles.

## Data Available Statement

The original contributions presented in the study are included in the article/supplementary material, and further inquiries can be directed to the corresponding author.

## Ethics Statement

The studies involving human participants were reviewed and approved by Ethics Committee of Shandong Mental Health Center. The patients/participants provided their written informed consent to participate in this study.

## Author Contributions

QL and BW were in charge of questionnaire design, statistical analysis, and writing. RZ and JW were in charge of data analysis and data entry. FS and GZ were in charge of the questionnaire survey. All authors designed this study and contributed to and approved the final manuscript.

## Funding

This work was supported by the Shandong Medical and Health Science and Technology Development Plan Project (2018ws296).

## Conflict of Interest

The authors declare that the research was conducted in the absence of any commercial or financial relationships that could be construed as a potential conflict of interest.

## Publisher’s Note

All claims expressed in this article are solely those of the authors and do not necessarily represent those of their affiliated organizations, or those of the publisher, the editors and the reviewers. Any product that may be evaluated in this article, or claim that may be made by its manufacturer, is not guaranteed or endorsed by the publisher.
